# Acute effects of butyrate on intestinal permeability in patients with irritable bowel syndrome assessed by a novel colonoscopy research model

**DOI:** 10.1080/19490976.2025.2545414

**Published:** 2025-08-14

**Authors:** Mathias W. Scharf, Richard A. Forsgård, Samira B. R. Prado, John-Peter Ganda Mall, Dirk Repsilber, Robert J. Brummer, Tatiana M. Marques, Rebecca Wall

**Affiliations:** a Nutrition-Gut-Brain Interactions Research Centre, School of Medical Sciences, Faculty of Medicine and Health, Örebro University, Örebro, Sweden; b Laboratory of Neuro-Immuno-Gastroenterology, Digestive System Research Unit, Vall d’Hebron Institut de Recerca (VHIR), Vall d’Hebron Hospital Universitari, Barcelona, Spain

**Keywords:** IBS, intestinal barrier function, *in vivo*, Ussing chamber

## Abstract

Irritable bowel syndrome (IBS) is a prevalent gastrointestinal disorder for which effective treatment strategies are insufficient. Butyrate, a microbiota-derived short-chain fatty acid believed to strengthen the intestinal barrier function, might be a potential new treatment option. This study aimed to investigate potential protective effects of acute *in vivo* butyrate exposure on intestinal barrier function in healthy subjects and patients with IBS. For this, we used an experimental colonoscopy-perfusion model for colon-specific butyrate delivery and adequate tissue sampling. Seventeen IBS and 17 healthy subjects underwent a colonoscopy procedure exposing a predefined colonic area to 100 mmol/L butyrate for 90 min *in vivo*. Mucosal biopsies collected pre- and post-butyrate exposure were stimulated in Ussing chambers with/without sodium deoxycholate (DC) to induce intestinal hyperpermeability. Intestinal permeability was measured by fluorescein isothiocyanate-dextran and horseradish peroxidase passage. DC-stimulation significantly increased para- and transcellular permeability in biopsies collected pre-butyrate exposure. DC-induced transcellular hyperpermeability was significantly alleviated in biopsies collected post-butyrate exposure compared to pre-exposure in patients with IBS (*p* = 0.034). In conclusion, we established a colonoscopy research model for colon-specific delivery and sampling and demonstrated acute protective effects of butyrate on transcellular intestinal permeability in patients with IBS. The results support butyrate’s potential role in novel treatment strategies in IBS. Clinicaltrials.gov number: NCT05249023

## GRAPHICAL ABSTRACT

**Figure:**
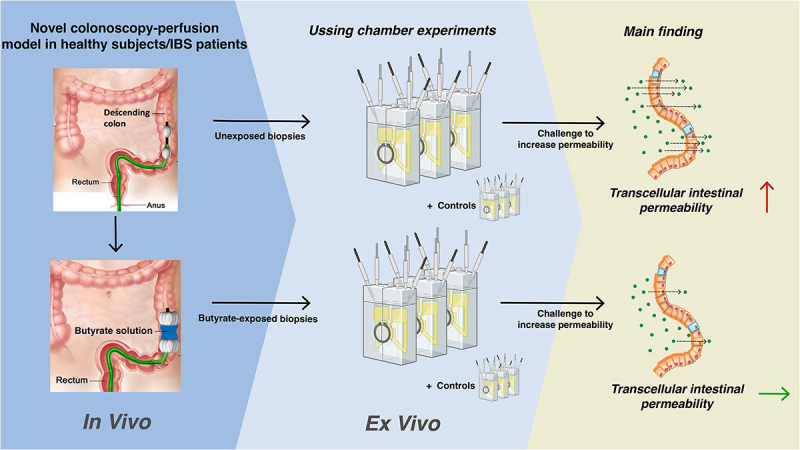

## Background

Irritable bowel syndrome (IBS) is one of the most common gastrointestinal disorders with a worldwide prevalence of 4.1%.^1^ IBS profoundly affects the patient’s quality of life and causes substantial economic costs due to medical consultations and absenteeism at the workplace.^2^ No treatment to date is considered universally applicable, and there is a high unmet need for novel treatment options for this debilitating disorder. The etiology of IBS is multifactorial and still not completely understood. However, pathophysiological mechanisms underlying the disorder are becoming clearer. These include visceral hypersensitivity, functional alterations in the gut-brain axis, dysregulated bowel motility, changes in intestinal microbiota composition and altered expression and release of mucosal and immune mediators.^3^ Additionally, a substantial proportion of patients with IBS show a dysfunctional intestinal barrier^4^ which is associated with abdominal pain^5^ and visceral hypersensitivity.^6^ Hence, the underlying mechanism might involve an impaired intestinal barrier and increased passage of bacterial components or food antigens across the intestinal wall, which could trigger a mucosal immune response leading to low-grade inflammation.^7,8^ The inhibition or restoration of intestinal barrier dysfuntion in patients or mice with post-infectious IBS has been shown to ameliorate visceral hypersensitivity^9^ and abdominal pain,^10^ highlighting the intestinal barrier function as a possible therapeutic target to improve the well-being of individuals living with IBS.

Butyrate is a short-chain fatty acid produced by microbial fermentation of mainly dietary fibers in the large intestine and is the preferred energy source of colonocytes. Additionally, butyrate exerts a broad range of functions and is considered an important regulator in intestinal homeostasis.^11^ Pre-clinical studies have demonstrated that butyrate can strengthen intestinal barrier function and decrease intestinal permeability^12–16^ by, for example, regulating the transcellular passage of bacteria^16^ and the expression of tight junction proteins.^17,18^ Vanhoutvin et al. showed that enemas with butyrate dose-dependently ameliorated visceral perception in healthy subjects.^19^ Additionally, patients with IBS have been shown to have reduced amounts of butyrate-producing bacteria,^20^ and colonic butyrate application^12^ or supplementation with the butyrate-producing bacteria *Faecalibacterium prausnitzii*^21^ significantly improved intestinal permeability and visceral perception in rat models of IBS. Furthermore, application of the butyrate-producing bacteria *Clostridium butyricum* or its metabolites by oral gavage protected mice from dextrane sulfate sodium (DSS)-induced disruption of the intestinal barrier function.^22^ These findings suggest that butyrate may offer a new treatment strategy to address intestinal barrier dysfunction and improve symptoms and well-being of patients with IBS. However, there is a translational gap and clinical data is largely missing. Only limited evidence exists to support the potential health effects of butyrate *in vivo* in humans and it remains unclear whether butyrate helps strengthen intestinal barrier function in patients with IBS. This is partly due to a lack of methods for colon-specific delivery and sampling, which are necessary for experimental designs of clinical studies to investigate hypotheses derived from animal and cell culture models.^23^ The objective of the present study was to assess whether acute exposure to butyrate *in vivo* protects the intestinal barrier against induced hyperpermeability in patients with IBS and healthy subjects. To address this objective, we established a novel experimental model for studying colonic mucosal biopsies obtained from the same subject before and after administration of butyrate directly into the human descending colon, targeting its site of action.

## Methods

### Study participants

A total of 34 participants, 17 healthy subjects, and 17 individuals with IBS (18–53 years old), were included in the study and underwent the study protocol. The selected number of 17 subjects per group was based on a t-test for independent group comparisons and unequal variances, significance level 5% and power 80%, considering a relevant difference to be at least as large as the standard deviation of the outcome. Participants were recruited via advertisements at Örebro University campus and on social media. Inclusion and exclusion criteria are listed in Table 1. Rome IV diagnostic criteria were used, and IBS subtypes were evaluated based on individual interviews. Gastrointestinal symptoms were assessed the day before and two days after the colonoscopy procedure using the Gastrointestinal Symptom Rating Scale – IBS (GSRS-IBS questionnaire; Supporting information). Among all study participants, one patient with IBS asked to stop the colonoscopy procedure due to pain.

**Table 1.** Inclusion and exclusion criteria.

Inclusion criteria	Exclusion criteria
∙ 18–65 years of age ∙ Signed informed consent ∙ Fulfilling Rome IV criteria (only for IBS patients)	∙ Known gastrointestinal disease ∙ Previous complicated gastrointestinal surgery (including e.g. cholecystectomy) ∙ Being pregnant or breast-feeding ∙ Alcohol or drug abuse ∙ Latex allergy ∙ Within the last 12 weeks before the colonoscopy: taking antibiotics, taking serotonin selective reuptake inhibitors (SSRI), taking serotonin norepinephrine reuptake inhibitors (SNRI) ∙ Within the last 4 weeks before the colonoscopy: regularly consuming probiotics, taking laxatives or anti-diarrhoeals ∙ Any clinically significant disease/condition which in the investigator’s opinion could interfere with the results of the trial

The study was approved by the Central Ethical Review Board of Sweden (Dnr 2016/464) and was registered with ClinicalTrials.gov, number NCT05249023. Throughout the study, the principles of the Helsinki Declaration were followed. All study participants received detailed information about the study and signed the informed consent before being enrolled in the study.

### Experimental model for controlled butyrate delivery and biopsy sampling in the human colon

**Figure 1.**
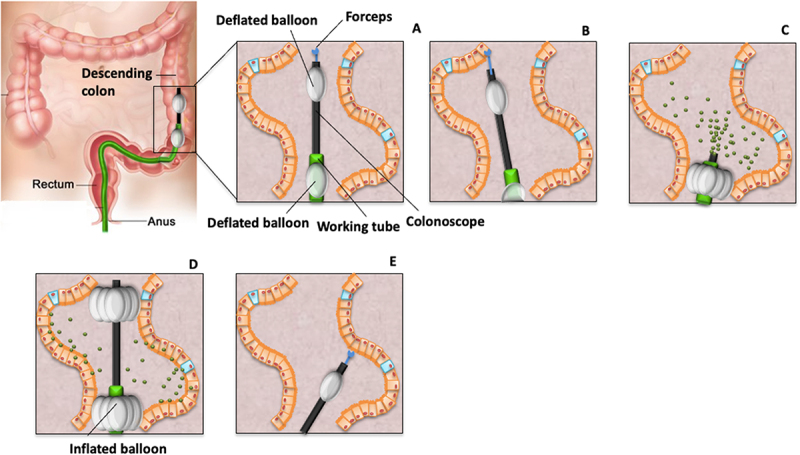
Experimental model used to collect colonic biopsies pre- and post-administration of butyrate in the human descending colon. This model uses double-balloon endoscopic equipment. Inflatable latex balloons are positioned at the tip of the working tube and the tip of the endoscope. Both balloons can be gently inflated to confine a well-defined area within the descending colon into which butyrate is administered. The figure shows the targeted area where butyrate is released (A), intestinal sampling before butyrate release (B), inflation of the distal balloon and butyrate release (C), inflation of the proximal balloon to ensure that the butyrate solution resides within the targeted area (D) and intestinal sampling post-exposure (E).

On the evening before the colonoscopy procedure, all study participants were instructed to consume a standardized low-fiber meal provided to them. The colonoscopy was performed in the unprepared bowel after 10 h of fasting and started between 8:00 and 9:00 am. Double-balloon endoscopic equipment was used, and the endoscope was fitted with an equally long working tube (TS-13140 Overtube, Fujifilm). Inflatable latex balloons (Balloon DBE, Fujifilm) were attached to the end of the working tube (distal balloon) and the tip of the endoscope (proximal balloon). In their final position in the descending colon, the two balloons were positioned approximately 20–25 cm apart from each other. Immediately after inflating the distal balloon to 25 mL volume, 60 mL of a room-tempered, sterile aqueous solution (Sterile water, Braun) containing 0.08% indigo carmine (Sigma-Aldrich) and 100 mmol/L sodium butyrate (PharmaGrade, SAFC Pharma) was released through the endoscope’s working channel. The working channel was afterward filled with an adequate amount (i.e., dead volume) of sterile water to avoid butyrate solution left in the channel. Immediately after the butyrate instillation, the proximal balloon was inflated to a volume of 15 mL to ensure that butyrate remained between the two inflated balloons within the targeted area throughout the entire exposure time. This defined area of the descending colon was exposed to the butyrate solution for 90 min. Before and after this 90-min exposure, mucosal biopsies were collected using a Captura biopsy forceps without spike (DBF-2.4–230, Cook Medical) and immediately transferred to ice-cold oxygenated modified Krebs-Ringer bicarbonate buffer (hereinafter called KRB; Supporting information). Biopsies obtained before the butyrate instillation were collected just proximally from the position of the proximal balloon, outside the defined exposure area. All biopsies were collected at the participants’ descending colon. The procedure is illustrated in Figure 1. Indigo carmine was added to the butyrate solution to better monitor its release and verify the collected biopsies’ exposure to butyrate. Indigo carmine is a blue food dye that is not absorbed by the mucosa and can be used in endoscopies to facilitate diagnosis and resections.^24^ Collected biopsies were transported in KRB buffer to the laboratory within 10 min and were immediately mounted in Ussing chambers.

### Ussing chamber experiments

A total of 12 biopsies were used per Ussing chamber experiment in triplicates per treatment. Six biopsies were collected prior to the *in vivo* exposure with butyrate (pre-exposure) and an additional six biopsies were collected after the 90 min *in vivo* exposure period (post-exposure). Among these two sets of biopsies collected, three biopsies were left unstimulated (Control), and three biopsies were stimulated with 1 mmol/L sodium deoxycholate (DC; Sigma-Aldrich), a secondary bile acid known to induce intestinal hyperpermeability.^25^

Biopsies were mounted in a randomized order in 1.5 mL Ussing chambers (Harvard Apparatus) and were held between two polyester films, exposing a round area of 1.77 mm^2^, as previously described.^26^ Fluorescein isothiocyanate-dextran (FITC-dextran) (3–5 kilodaltons, Sigma-Aldrich) and 45 kilodaltons horseradish peroxidase (HRP; Sigma-Aldrich) were used as paracellular and transcellular permeability markers, respectively. The Ussing method, the measurement of transepithelial resistance (TER), short circuit current (I_SC_), potential difference (PD), and the measurement of FITC-dextran and HRP concentrations are described in detail in the Supporting information. FITC-dextran and HRP passages are expressed as differences in their concentrations 60 min after adding the permeability markers (time point 60 min, T60) compared to the start (time point 0 min, T0). TER was measured in Ω*cm^2^ throughout the experiment and TER reduction is given as % decrease in TER from the start (T0).

### Study endpoints

The primary endpoint of the study was to determine whether an acute *in vivo* exposure to butyrate alters intestinal permeability, as assessed *ex vivo* in Ussing chamber experiments, with or without DC-stimulation. Permeability was measured by FITC-dextran or HRP passage in Ussing chamber experiments with mucosal biopsies collected from individuals with IBS and healthy subjects. Secondary outcomes were as follows: 1) to assess adverse effects of the established colonoscopy-perfusion model on IBS-related symptoms evaluated by GSRS-IBS questionnaires; 2) to evaluate the effects of the acute butyrate exposure on electrophysiological parameters measured in Ussing chamber experiments (TER and I_sc_) in patients with IBS and in healthy subjects; 3) to determine the effect of DC on intestinal permeability (FITC-Dextran and HRP passage) in healthy subjects and in patients with IBS and 4) to examine differences in baseline permeability between patients with IBS and healthy subjects.

### Statistical analysis

Differences between treatment groups in FITC-dextran and HRP passages were analyzed after log-transformation with a linear mixed effects model (Restricted maximum likelihood, REML) with Geisser-Greenhouse correction and Tukey’s multiple comparison test. Butyrate exposure (pre- or post-exposure), DC treatment (DC-stimulated or control) and participant group (healthy or IBS) were handled as fixed effects, while subjects were treated as random effects. Changes in TER reduction between the treatment groups in healthy subjects and patients with IBS were analyzed the same way but without prior log-transformation. Differences between DC-effect pre-exposure vs post-exposure in delta values of FITC-dextran and HRP passages were tested using Friedman test with Dunn’s multiple comparison test. One value in this dataset was missing due to technical reasons (DC-effect, HRP, post-exposure, IBS) and was replaced with the same value it was statistically compared to (DC-effect, HRP, pre-exposure, IBS), choosing a conservative approach by assuming butyrate had no effect. To test whether the DC-effect on TER pre-exposure was different from post-exposure in healthy subjects or individuals with IBS, a linear mixed effects model (REML) with Geisser-Greenhouse correction and Sidak’s multiple comparison correction was used. Differences in DC-effect on TER between patients with IBS and healthy subjects were analyzed the same way. Differences between healthy subjects and patients with IBS in baseline intestinal permeability (Control, pre-exposure) were analyzed using Kruskal–Wallis test with Dunn’s multiple comparison test. The comparison of TER for control biopsies pre-exposure vs post-exposure in healthy subjects and patients with IBS was performed with Wilcoxon signed rank tests and Bonferroni multiple comparison correction. Differences in intestinal permeability between male and female study participants were tested by ordinary 2-way ANOVA.

Normality and log-normality were tested using the Shapiro–Wilk test. Differences were considered significant at *p* < 0.05. All statistical analyses were performed using GraphPad Prism 9.3.1.

## Results

### Study participant characteristics and GSRS-IBS scores

Characteristics of all study participants are listed in Table 2. GSRS-IBS scores were evaluated to monitor for potential adverse effects associated with the established colonoscopy-perfusion model. Average GSRS-IBS scores of healthy subjects were significantly lower compared with scores of patients with IBS before the colonoscopy procedure and 2 days after. There was no significant difference between total scores or subscores before the colonoscopy procedure compared with two days after the procedure.

**Table 2.** Characteristics of study participants and GSRS-IBS questionnaire scores.

Characteristic	Healthy subjects	IBS patients	IBS-D	IBS-C	IBS-M
Number	17	17	9 (53%)	3 (18%)	5 (29%)
Sex (Male/Female)	12/5	4/13	3/6	1/2	0/5
Age (years ± SD)	30.2 ± 9.0	31.8 ± 9.9	30.3 ± 8.0	24.7 ± 8.3	38.8 ± 11.3
Medical characteristics and comorbidity					
Appendectomy	1	0			
Asthma	1	0			
Eczema	0	2			
Lactose intolerance	0	2			
Secondary amenorrhea	0	1			
Grown-up congenital heart disease	0	1			
Hashimoto	0	1			
Hypermobile Ehlers-Danlos Syndrome	0	1			
Medications/Supplementations within last 4 weeks before the colonoscopy					
Food supplementation (Iron, Folic acid, Vit B12, L-Arginin, Creatin)	0	4			
Dimetakon	0	1			
Ceterizin	0	1			
Levaxin	0	1			
GSRS-IBS scores before colonoscopy (mean ±SD)					
Total GSRS-IBS score	1.4 ± 0.4 ****	3.8 ± 0.8	3.5 ± 0.8	3.8 ± 0.4	4.4 ± 0.9
Diarhoea subscore	1.6 ± 0.7 ****	3.6 ± 1.3	3.9 ± 1.1	2.9 ± 1.7	3.5 ± 1.4
Constipation subscore	1.4 ± 0.7 ****	3.6 ± 1.7	2.9 ± 1.4	3.9 ± 2.5	4.9 ± 1.1
Bloating subscore	1.6 ± 0.6 ****	4.7 ± 1.4	4.0 ± 1.5	5.2 ± 0.8	5.7 ± 0.8
Abdominal pain subscore	1.4 ± 0.5 ****	4.0 ± 0.9	4.0 ± 1.1	4.2 ± 0.3	4.0 ± 1.1
Satiety subscore	1.1 ± 0.4 ****	2.9 ± 1.8	2.6 ± 1.5	2.8 ± 1.8	3.4 ± 2.4
GSRS-IBS scores 2 days after colonoscopy (mean ±SD)					
Total GSRS-IBS score	1.6 ± 0.5 ****	3.3 ± 0.7	2.9 ± 0.5	3.6 ± 0.8	3.9 ± 0.5
Diarhoea subscore	1.7 ± 0.7 ***	3.1 ± 1.2	3.3 ± 1.2	2.7 ± 2.1	3.1 ± 0.7
Constipation subscore	1.4 ± 0.5 ****	3.0 ± 1.7	2.3 ± 1.3	3.8 ± 2.8	3.9 ± 1.1
Bloating subscore	1.8 ± 0.7 ****	4.2 ± 1.4	3.2 ± 0.9	5.0 ± 1.2	5.5 ± 0.6
Abdominal pain subscore	2.0 ± 0.9 ****	3.7 ± 1.0	3.3 ± 1.0	4.5 ± 1.0	3.9 ± 1.0
Satiety subscore	1.3 ± 0.4 *	2.4 ± 1.4	2.2 ± 1.4	2.0 ± 0.9	2.8 ± 1.8

*p < 0.05, ***p < 0.001, ****p < 0.0001 compared to the respective (sub)score in patients with IBS.

### Butyrate exposure in vivo protected colonic biopsies of individuals with IBS against DC-induced hyperpermeability ex vivo

**Figure 2.**
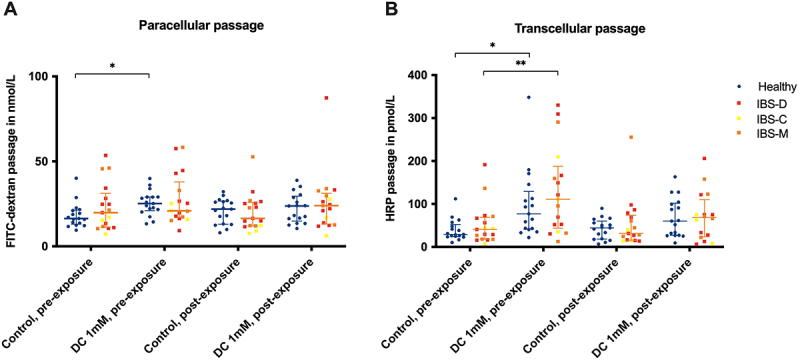
Para- and transcellular permeability marker passage in colonic biopsies obtained from healthy subjects (blue circles) and patients with IBS (red, orange and yellow squares) pre- and post-butyrate exposure in vivo. Before exposure (pre-exposure) and 90 minutes after the in vivo exposure (post-exposure) to 100 mmol/L butyrate, colonic biopsies were left unstimulated (control) or stimulated with 1 mmol/L sodium deoxycholate (DC). Concentrations are shown with indicated median and interquartile range. *p < .05, **p < .01. n_Healthy_ = 17, n_IBS_ = 17.

Intestinal permeability was assessed in colonic biopsies before and after *in vivo* butyrate exposure, using the permeability markers FITC-dextran and HRP. TER was used to reflect paracellular barrier integrity. At baseline (Control, pre-exposure), healthy subjects and patients with IBS (all IBS-subtypes included or separated by subtype) did not show significant differences in the passage of permeability markers or TER (Supporting information, Table S1). Furthermore, both in patients with IBS and healthy subjects, there was no significant difference between unstimulated control biopsies pre-exposure compared to post-exposure of butyrate in FITC-dextran passage, HRP passage, or TER (Supporting information, Table S2). Compared to unstimulated controls, DC-stimulated biopsies showed significantly increased passage of both FITC-dextran (*p* = 0.015, Figure 2(A)) and HRP (*p* = 0.027, Figure 2(B)) in healthy subjects pre-exposure but not post-exposure to butyrate. In patients with IBS, passage of HRP was significantly higher in DC-stimulated biopsies compared to unstimulated control pre-exposure (*p* = 0.004) but not post-exposure (Figure 2). The FITC-dextran passage in biopsies collected from individuals with IBS did not significantly differ between DC-stimulated biopsies and unstimulated control biopsies both pre-exposure and post-exposure. No significant differences in permeability marker passage between female and male study participants were observed (*p* = 0,11 in FITC data and *p* = 0,47 in HRP data).

**Figure 3.**
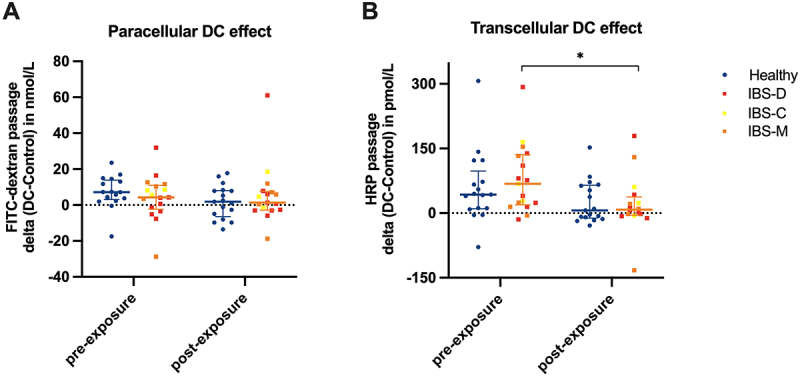
Effects of sodium deoxycholate on human colonic biopsies of healthy subjects (blue circles) and patients with IBS (red, orange and yellow squares) before and after in vivo butyrate exposure. Delta values of FITC-dextran and HRP passage (DC stimulated biopsy – control biopsy) are depicted to elucidate butyrate’s effect on DC-induced hyperpermeability. Delta values are shown with indicated median and interquartile range. *p < .05. n_Healthy_ = 17, n_IBS_ = 17.

To elucidate whether butyrate exposure *in vivo* affected DC-induced hyperpermeability, we compared DC-effects pre-exposure vs post-exposure (Figure 3). DC-effects were calculated as a delta by subtracting FITC-dextran or HRP passage of control biopsies from FITC-dextran or HRP passage of DC-stimulated biopsies, respectively. In patients with IBS, the transcellular DC-effect was significantly lower post-exposure compared to pre-exposure to butyrate (*p* = 0.034, Figure 3(B)). No significant differences were observed in healthy subjects. All DC-effect delta values and DC-effect rates can be found in Table 3.

**Table 3.** DC-effect on permeability marker passage in biopsies collected from healthy subjects and patients with IBS pre- and post-exposure to butyrate *in vivo*..

	Healthy subjects		IBS patients	
Measurement	Pre-exposure	Post-exposure	Pre-exposure	Post-exposure
Paracellular DC-effect (nmol/L)	7.1 [3.0–13.8)	1.8 [−6.5–8.1)	4.3 [−2.4–11.0]	1.4 [−2.8–7.5]
Transcellular DC effect (pmol/L)	43.1 [10.2–97.5]	6.3 [−11.6–64.7]	67.9 [19.3–135.7]	10.1 [−4.1–41.3]*
Paracellular DC effect rate (%)	37.8 [19.4–96.3]	8.6 [−21.9–40.9]	29.9 [−12.5–54.4]	6.9 [−16.1–38.7]
Transcellular DC effect rate (%)	107.3 [37.7–308.2]	12.8 [−29.9–103.2]	113.1 [82.7–197.9]	9.0 [−13.2–59.1]*

Before and after in vivo butyrate exposure for 90 minutes colonic biopsies were collected, mounted in Ussing chambers and passage of paracellular and transcellular permeability markers, FITC-dextran and HRP respectively, was measured. DC-effect on FITC-dextran and HRP passage was calculated as delta values of passage (DC stimulated biopsy minus control biopsy). DC-effect rates are calculated as the DC-effect divided by the permeability marker passage of the control biopsies and given. All values except for P-values are given as median with interquartile range. *p < 0.05 compared to pre-exposure in the same measurement group. n_Healthy_ = 17, n_IBS_ = 17.

### Electrophysiological parameters of colonic biopsies

Transepithelial resistance (TER) was measured throughout the Ussing experiments. Relative reduction in TER at the end (T60) compared to the beginning of each experiment (T0) was calculated as percentage (Table 4). In healthy subjects, DC-stimulated biopsies showed a significantly larger TER reduction in both pre- and post-exposure compared to control biopsies. In patients with IBS there was no significance, both pre-exposure and post-exposure. The DC-effect on TER (difference in TER reduction between DC- and control group) was not significantly different pre-exposure compared to post-exposure both in IBS and healthy. Additionally, there was no significant difference in DC-effect on TER between IBS and healthy, both pre- and post-exposure. Baseline I_SC_ (Unstimulated Control, T0) was significantly higher post-exposure compared to pre-exposure to butyrate in healthy subjects, but not in patients with IBS (Supporting information, Table S3).

**Table 4.** Reduction in transepithelial resistance (TER) of colonic biopsies in percentage (mean with standard deviation).

Treatment group	Healthy	IBS
Control, pre-exposure	13.78 ± 4.75	13.15 ± 7.83
DC 1 mM, pre-exposure	23.87 ± 9.81 **	19.54 ± 6.25
Control, post-exposure	12.54 ± 8.38	13.28 ± 7.96
DC 1 mM, post-exposure	22.51 ± 9.65 **	22.46 ± 7.28

**p < 0.01 compared to control group at the same timepoint (pre- or post-exposure). n_healthy_ = 17, n_IBS_ = 17.

## Discussion

Despite a plethora of pre-clinical evidence regarding butyrate’s beneficial effects on colonic health, data from human studies to substantiate these effects are still scarce. A large body of evidence highlights butyrate’s importance for maintaining intestinal homeostasis and studies mostly from *in vitro* and animal models verify its strengthening effects on intestinal barrier function.^12,14,27^ However, translational approaches to study butyrate’s effects on intestinal barrier function in humans are required, especially for the potential to use butyrate as a clinical treatment tool for barrier dysfunction. In this study, we assessed the effect of an acute butyrate exposure *in vivo* on intestinal permeability in healthy subjects and individuals living with IBS. For this, we established a novel experimental endoscopic procedure that allows the collection of mucosal biopsies from the same subject before and after a controlled *in vivo* exposure to butyrate in the human descending colon without prior bowel cleansing.

In the present study, we observed a significantly reduced stressor permeability effect (DC-effect) in biopsies collected from patients with IBS post-butyrate exposure compared with biopsies collected pre-exposure. This suggests an acute protective effect of butyrate on intestinal barrier function in patients with IBS. To our knowledge, this is the first time that a short-time *in vivo* exposure to butyrate in the distal colon of individuals with IBS showed protective effects on intestinal barrier function. Our findings further substantiate previous insights from animal models for IBS demonstrating butyrate’s protective effect on intestinal permeability.^12,22^

Butyrate’s protective effect on intestinal permeability was observed only transcellularly (HRP passage) but not paracellularly (FITC-dextran passage and TER). This is in line with an *in vitro* study from Lewis et al. in which butyrate (3 mmol/L and 50 mmol/L) reduced the translocation of *Escherichia coli* across monolayers of metabolically stressed colon-derived epithelial cell lines, while the provoked reduction in TER remained unaffected by butyrate.^16^ The authors postulated that bacteria crossed the epithelium mainly transcellularly, with butyrate having a selective protective effect on that route. Additionally, Bednarska et al. found increased translocation of *E. coli* and *Salmonella typhimurium* across biopsies from patients with IBS compared to healthy subjects and detected only transcellular bacterial passage.^4^ However, butyrate’s mechanism of action should be investigated further as it is unknown whether butyrate affects bacterial translocation *in vivo*. Additionally, the *in vivo* butyrate exposure only influenced intestinal permeability when the barrier function was challenged with DC. Butyrate did not affect the participants’ baseline intestinal permeability. This indicates that butyrate might not *per se* strengthen the intestinal barrier, but rather makes it more resilient against perturbation. In contrast to this observation, cell culture studies have shown that butyrate alone (without any challenge) can decrease paracellular permeability, for example by affecting tight junction protein expression and assembly.^14,17^ This could be due to a disparity between *in vitro* and *in vivo* approaches: the stimulation with butyrate *in vivo* in the human colon with its heterogenous and complex cellular interactions and an intact mucous layer may differ from a direct exposure on more homogenous cell monolayers. Another explanation could be that changes in gene expression patterns observed in *in vitro* studies require continuous, long-term exposure to butyrate. The 90-min exposure time applied in the present study might have been insufficient to induce these changes. Our study suggests that, independent of effects on baseline intestinal permeability, a beneficial effect of butyrate in IBS might lie in protecting the intestinal barrier function when it is under challenge.

Previous research has shown that patients with IBS have an increased permeability of the large intestine (both paracellular and transcellular) compared with healthy subjects^4,5^ A systematic review by Hanning et al. (2021) thoroughly presented the body of evidence supporting intestinal barrier dysfunction in patients with IBS, being specifically frequent in those with IBS-D.^28^ Baseline permeability marker passage and TER results from this study, separated by IBS-subtype or including all subtypes, do not coincide with these findings but are in line with several studies included in the systematic review which did not observe an increased intestinal permeability in patients with IBS. Additionally, we did not find differences in the baseline I_SC_ of patients with IBS compared to healthy subjects, in concordance with a previous study on IBS-C patients.^29^ Of note, the number of individuals for each IBS-subtype in our study is relatively small. This might have been a reason why previous research demonstrating an increased intestinal baseline permeability in patients with IBS compared to healthy subjects were not observed in the present study. Additionally, previous research observed sex-associated differences in intestinal permeability.^30^ We did not observe differences between female and male study participants in our intestinal permeability data. A larger number of study participants would have improved the study’s ability to detect both baseline permeability differences between healthy subjects and patients with IBS and potential sex-associated effects. However, to exclude an influence of a different sex distribution between the participant groups, matching the number of male and female participants would be preferable in future studies on intestinal permeability. We could, however, detect a significant increase in net ion transport (measured as I_SC_) across the biopsies of healthy subjects, but not individuals with IBS, after butyrate exposure. The precise mechanism of this remains unclear.

In this study, we used DC to induce hyperpermeability in colonic biopsies *ex vivo*. This secondary bile acid is a potent agent inducing intestinal barrier dysfunction^31^ with increased paracellular and transcellular intestinal permeability.^25,32^ Our data on permeability and TER reduction support these observations. Stimulation with DC exerted a substantial increase in transcellular permeability marker passage both in patients with IBS and in healthy subjects. However, the paracellular effect of DC, measured as FITC-dextran passage and TER reduction, is less pronounced and hence inferred a reduced potential to detect differences between patients with IBS and healthy subjects as well as between pre- and post-exposure to butyrate. It should be noted that while DC-induced hyperpermeability does not fully reflect the complex and multifactorial nature of intestinal permeability alterations in patients with IBS, it provides a valuable experimental model for studying mechanisms of barrier dysfunction.

The novel colonoscopy-perfusion model implemented in this study was developed to overcome the lack of methods in translational research in gastroenterology that allow controlled and colon-specific delivery of substances and adequate sampling pre- and post-exposure. The established colonoscopy model supports a broad variety of experimental designs and can be used with other substances of interest, in other patient groups, and with a range of possible downstream analyses of collected samples such as mucosal biopsies and blood. The availability of colonic biopsies from patients before and after standardized modifications of the environment in the human colon, without the need for bowel cleansing, makes the model especially valuable for translational approaches in clinical research. The Ussing chamber technique was chosen to enable a standardized and mechanistic investigation of butyrate’s effect on intestinal permeability by comparing butyrate-exposed versus unexposed colonic tissue from the same participant, time and colonic location. This method also enabled the distinction between paracellular and transcellular permeability and the possibility to apply a controlled stimulation with DC to induce intestinal permeability.

The endoscopic procedure showed good applicability in the chosen study population and did not induce or exacerbate gastrointestinal problems evaluated by GSRS-IBS scores. It should be noted that symptom assessments using GSRS-IBS were conducted to monitor for adverse effects related to the established colonoscopy model and not intended to evaluate the efficacy of the butyrate exposure. The selected 90 min *in vivo* intervention used was chosen to be within an acceptable and tolerable time frame for the participants. Hence, this experimental model is suitable to study acute but not long-term modifications of the intestinal environment.

Intestinal barrier dysfunction is a substantial element of IBS pathophysiology and has been linked to symptoms like abdominal pain^5^ and visceral hypersensitivity.^6^ Increasing the resilience against perturbations of the intestinal barrier, as demonstrated in this study by acute exposure of the colonic mucosa to butyrate, might be a way to ameliorate symptoms and increase the well-being of individuals living with IBS. In a rat model of IBS, the application of butyrate has already been shown to strengthen the intestinal barrier and attenuate visceral hypersensitivity.^12^ Additionally, microencapsulated sodium butyrate could reduce the frequency of abdominal pain in individuals with IBS.^33^ Our study adds valuable mechanistic data *in vivo* in humans and substantiates butyrate’s potential as part of novel treatment approaches in patients with IBS. It is important to acknowledge that the novel colonoscopy-perfusion model established in this study is not intended for clinical application. The 90-min colonic butyrate exposure, while effective for experimental purposes, is not a feasible treatment modality. Our study was designed to examine acute, localized effects of butyrate and serves primarily as a translational tool to evaluate direct effects of bioactive compounds on the human colonic mucosa under controlled conditions. The findings provide a rationale for further investigation into more feasible delivery strategies, such as dietary interventions (using e.g. high-fiber diets), encapsulated formulations, or other microbiota-targeted approaches that enhance endogenous butyrate production. Noteworthy, the selected butyrate concentration of 100 mmol/L used in the present study can be achieved intraluminally in the human colon during a high-fiber diet and has previously been used in several studies.^19,34–36^ A controlled and effective manipulation of the luminal butyrate levels, however, is still challenging and not yet clinically feasible. Furthermore, our study does not imply a sufficient evaluation of IBS symptoms, limiting its clinical relevance. Future studies should investigate different ways to manipulate luminal butyrate concentrations (e.g. through diet, probiotics, prebiotics or administration of encapsulated sodium butyrate) to confirm their effect on the intestinal barrier function in individuals with IBS. Measurements of symptom severity and quality of life should be included to assess the clinical relevance of butyrate’s effects. Besides that, butyrate is part of a complex interplay of environment, intestinal microbiota and the human host, implicating that a successful therapeutic approach is likely not found through butyrate’s effects alone, but rather in addressing a combination of different key components of this network.

## Conclusion

We established a novel colonoscopy-perfusion procedure for colon-specific delivery of bioactive substances and adequate sampling of mucosal biopsies pre- and post-exposure *in vivo*. Applying this research tool, we showed acute protective effects of *in vivo* butyrate exposure on the transcellular pathway of intestinal barrier function in patients with IBS.

## Supplementary Material

Supplementary material Scharf et al 2nd revision.docx

## Data Availability

Original data presented in this article can be requested under https://doi.org/10.60689/jxc0-3238.
