# Supplementary material for: Acute effects of butyrate on intestinal permeability in patients with irritable bowel syndrome assessed by a novel colonoscopy research model
Source: Gut Microbes. 2025 Aug 14;17(1):2545414. doi: 10.1080/19490976.2025.2545414 (PMC12355679; doi:10.1080/19490976.2025.2545414)
Supplement: Supplementary material Scharf et al 2nd revision.docx [file KGMI_A_2545414_SM8524.docx]

Supporting information

Methods

*Gastrointestinal Symptom Rating Scale - IBS (GSRS-IBS) questionnaire*

On the day before the colonoscopy procedure and on the second day after the procedure, study participants completed the GSRS-IBS questionnaire, a validated 13-items questionnaire characterising problems with abdominal pain, satiety, diarrhoea, constipation and bloating ^1^. The GSRS-IBS results were statistically analysed using a repeated measurements 2-way ANOVA with Sidak’s multiple comparisons test to compare scores of healthy subjects with scores of patients with IBS. Friedman test with Dunn’s multiple comparisons test was used to compare scores before the colonoscopy vs scores 2 days after the colonoscopy, in patients with IBS and in healthy subjects separately.

*Ussing chamber experiments*

Immediately after mounting a biopsy in the Ussing chamber, both sides of the chamber were filled with ice-cold modified Krebs-Ringer bicarbonate buffer (aqueous solution with 115 mmol/L NaCl, 1.25 mmol/L CaCl_2_, 1.2 mmol/L MgCl_2_, 2 mmol/L KH_2_PO_4_ and 25 mmol/L NaHCO_3_, set to a pH of 7.2 with 1 mol/L hydrochloric acid-solution and then oxygenated with gas containing 95% O_2_ and 5% CO_2_, from now on called KRB). The buffer on the mucosal side contained mannitol (0.01 mol/L) and the buffer on the serosal side contained glucose (0.01 mol/L). The biopsies were provided oxygen via constant oxygenation with 95% oxygen/5% carbon dioxide-gas mix throughout the experiment. Additionally, the chambers were held at 37 °C. To monitor tissue viability, the electrophysiological parameters transepithelial resistance (TER), potential difference (PD) and short circuit current (I_SC_) were measured every 30 seconds throughout the experiment. Biopsies with a PD > 0.5 were excluded because of uncertain tissue viability ^2^. Biopsies were equilibrated in the Ussing chambers for a total of 40 minutes, whereas the buffers on both sides were exchanged with 37 °C warm KRB containing mannitol/glucose after 10 and 20 minutes. After 40 minutes of equilibrium time, sodium deoxycholate (DC) and the two permeability markers, fluorescein isothiocyanate-dextran (FITC-dextran) 3-5 kilodaltons (Sigma-Aldrich, St. Louis, MO, USA) and 45 kilodaltons horseradish peroxidase (HRP; Sigma-Aldrich) were added to the mucosal side of the chambers. DC had a final concentration of 1 mmol/L, FITC-dextran of 2.5 nmol/L and HRP of 5.34 µmol/L in the chamber. Permeability marker passage was assessed at start (T0) and 60 minutes after the permeability markers were added (T60). Samples were collected from the serosal side and FITC-dextran concentration was determined by fluorescence measurement at λ_ex_=485 nm and λ_em_=530 nm using a Cytation 3 multimode reader (BioTek). Horseradish peroxidase was measured by ELISA using the QuantaBlu Fluorogenic Peroxidase Substrate Kit (Thermo Fisher Scientific, USA) as previously described ^3^.

I_SC_ was statistically analysed as follows: To test whether baseline I_SC_ (Unstimulated Control, T0) pre-exposure to butyrate was different from post-exposure and whether there are differences between patients with IBS and healthy subjects we used a linear mixed effects model (Restricted maximum likelihood, REML) with Sidak multiple comparisons test. Butyrate exposure and participant group were handled as fixed effects and subjects were handled as random effects.

Results

Table S1: Baseline comparison of FITC-dextran passage, HRP passage and TER values in healthy subjects and IBS patients.

| Measurement | Healthy subjects | IBS patients | P-value |
| --- | --- | --- | --- |
| FITC-dextran passage (nmol/L) | 16.35 [13.07-21.37] | 19.85 [11.36-31.21] | 0.88 |
| HRP passage (pmol/L) | 29.14 [24.17-52.43] | 40.74 [17.91-68.97] | >0.99 |
| TER (Ω*cm2) | 19.87 [18.64-22.80] | 19.69 [17.65-23.70] | >0.99 |

Before in vivo butyrate exposure, colonic biopsies were collected, mounted in Ussing chambers and measurements were run without DC-stimulation (Control, pre-exposure). No significant differences on baseline intestinal permeability between healthy subjects and IBS patients were detected. Data of one IBS patient is missing due to technical problems. All values except for P-values are given as median with interquartile range. n_Healthy_=17, n_IBS_=16

Table S2: Comparison of unstimulated control biopsies pre-exposure compared to post-exposure to butyrate *in vivo* in healthy subjects and patients with IBS.

|  | Healthy subjects | | | |  | | IBS patients | | |
| --- | --- | --- | --- | --- | --- | --- | --- | --- | --- |
| Measurement | Pre-exposure | Post-exposure | P-value |  | | Pre-exposure | | Post-exposure | P-value |
| FITC-dextran passage (nmol/L) | 16.35 [13.07-21.37] | 21.93 [13.05-26.65] | 0.95 |  | | 19.85 [11.36-31.21] | | 16.48 [12.07-25.85] | 0.99 |
| HRP passage (pmol/L) | 29.14 [24.17-52.43] | 44.46 [18.46-60.15] | >0.99 |  | | 40.74 [17.91-68.97] | | 31.46 [15.83-73.87] | >0.99 |
| TER (Ω*cm2) | 19.87 [18.64-22.80] | 18.85 [16.65-22.04] | 0.13 |  | | 19.69 [17.65-23.70] | | 21.30 [15.33-23.31] | >0.99 |

Before and after in vivo butyrate exposure for 90 minutes colonic biopsies were collected, mounted in Ussing chambers, and FITC-dextran passage, HRP passage and TER were measured. P-values compare pre-exposure vs post-exposure in healthy subjects and in patients with IBS, reflecting the effect of in vivo butyrate exposure on the intestinal permeability of unstimulated biopsies. No significant differences were detected. TER data of one IBS patient is missing due to technical problems. All values except for P-values are given as median with interquartile range. n_Healthy_=17, n_IBS_=17, n_IBS_=16 in TER measurement

The I_SC_ represents net ion flux across the colonic biopsies mounted in Ussing chambers. Baseline I_SC_ was significantly higher post-exposure compared to pre-exposure to butyrate in healthy subjects (p=0.015), but not in patients with IBS (p=0.40), or in the different IBS subtypes (p=0.100 for IBS-D, p>0.98 for IBS-C and IBS-M). Additionally, there was no significant difference in baseline I_SC_ between healthy and IBS patients (Table S4).

Table S3: Baseline short circuit current (I_SC_) in patients with IBS and healthy subjects pre- and post-exposure to butyrate.

| Group | Pre-exposure | Post-exposure |
| --- | --- | --- |
| Healthy | 62.9 ± 14.1 | 83.0 ± 27.1 * |
| IBS | 73.3 ± 30.6 | 81.7 ± 26.6 |
| IBS-D | 63.3 ± 15.3 | 85.1 ± 22.6 |
| IBS-C | 95.4 ± 56.3 | 92.2 ± 39.8 |
| IBS-M | 75.9 ± 30.8 | 69.9 ± 26.1 |

Before and after in vivo butyrate exposure for 90 minutes, colonic biopsies were collected, mounted in Ussing chambers and I_SC_ was measured. Baseline I_SC_ was significantly higher post-exposure compared to pre-exposure in healthy subjects (p=0.015), but not in patients with IBS (p=0.40). There was no significant difference in baseline I_SC_ in healthy compared to IBS patients. I_SC_ values are given in µA/cm^2^. Data of one participant (IBS-D) is missing due to technical problems. *p<0.05 compared to pre-exposure in the same group. n_Healthy_=17, n_IBS_=16 (of which 8 IBS-D, 3 IBS-C and 5 IBS-M)

1. Wiklund IK, Fullerton S, Hawkey CJ, et al. An irritable bowel syndrome-specific symptom questionnaire: development and validation. Scand J Gastroenterol 2003;38:947-54.

2. Wallon C, Braaf Y, Wolving M, et al. Endoscopic biopsies in Ussing chambers evaluated for studies of macromolecular permeability in the human colon. Scand J Gastroenterol 2005;40:586-95.

3. Ganda Mall JP, Casado-Bedmar M, Winberg ME, et al. A beta-Glucan-Based Dietary Fiber Reduces Mast Cell-Induced Hyperpermeability in Ileum From Patients With Crohn's Disease and Control Subjects. Inflamm Bowel Dis 2017;24:166-178.
